# Association between tonsillectomy plus steroid pulse therapy and renal outcomes in patients with IgA nephropathy: a retrospective cohort study

**DOI:** 10.1186/s12882-025-04408-5

**Published:** 2025-08-23

**Authors:** Ayano Hayashi, Kayoko Mizuno, Kanna Shinkawa, Kazunori Sakoda, Satomi Yoshida, Masato Takeuchi, Motoko Yanagita, Koji Kawakami

**Affiliations:** 1 Department of Pharmacoepidemiology, Graduate School of Medicine and Public Health, Kyoto University, Yoshida-Konoe-Cho, Kyoto, Sakyo-Ku 606-8501 Japan; 2 Department of Nephrology, Graduate School of Medicine, Kyoto University, Kyoto, Japan; 3 Department of Digital Health and Epidemiology, Graduate School of Medicine and Public Health, Kyoto University, Kyoto, Japan; 4 Division of Social Medicine, Department of Clinical Medicine, Institute of Medicine, University of Tsukuba, Ibaraki, Japan; 5 Graduate School of Public Health, Shizuoka Graduate University of Public Health, Shizuoka, Japan; 6 Institute for the Advanced Study of Human Biology (WPI-ASHBi), Kyoto University, Kyoto, Japan

**Keywords:** IgA nephropathy, Healthcare record database, Retrospective cohort study, Steroid pulse, Tonsillectomy

## Abstract

**Background:**

IgA nephropathy (IgAN) is the most common disorder in chronic glomerulonephritis, and various treatment methods have been established. Tonsillectomy and steroid pulse therapy (TSP) are widely performed in Japan. However, their correlation with renal outcomes remains unclear. In this study, we aimed to examine the association between renal prognosis and steroid pulse therapy with or without tonsillectomy.

**Methods:**

In this retrospective cohort study, we identified patients diagnosed with IgAN between April 2002 and March 2021 using a Japanese healthcare records database. We selected patients with a prescription history of methylprednisolone for three consecutive days within one year of diagnosis and an estimated glomerular filtration rate (eGFR) of 30 mL/min/1.73 m^2^ or above. We categorized the patients into TSP and steroid pulse therapy (SP) groups based on whether they underwent tonsillectomy within one year of diagnosis. The primary outcome was the composite outcome of a 30% decline in eGFR and initiation of dialysis, and the secondary outcomes comprised the composite outcome of end-stage renal failure and initiation of dialysis and eGFR slope. Inverse probability of treatment weighting (IPTW) using propensity score, Kaplan–Meier survival analysis, and weighted Cox regression analysis by applying IPTW were performed.

**Results:**

Overall, 550 patients were eligible for the main analysis, and approximately 40% underwent tonsillectomy within 1 year of IgAN diagnosis: 221 in the TSP group and 329 in the SP group. The primary outcome did not differ between the groups (hazard ratio, 0.58; [95% confidence interval: 0.22–1.54]; P = 0.28). The groups did not differ in terms of the secondary outcomes.

**Conclusions:**

Although we could not demonstrate the effectiveness of TSP on renal prognosis in patients with IgAN, this study may have been underpowered, and there are certain limitations due to the information available from the database, we were able to evaluate the association of TSP on renal outcomes using real-world data.

**Clinical Trial Number:**

Not applicable.

**Supplementary information:**

The online version contains supplementary material available at 10.1186/s12882-025-04408-5.

## Background

IgA nephropathy (IgAN) is the most common disease in chronic glomerulonephritis (CGN)[1], with an estimated 30–40% of patients progressing to end-stage renal failure (ESRD) within 20 years of diagnosis [2]. In Japan, CGN is the second most common underlying cause among chronic dialysis patients, accounting for approximately 24% of the cases [3]. Among the various treatment approaches established for IgAN, such as steroid therapy, immunosuppressive agents, renin-angiotensin (RAS) inhibitors, antiplatelet drugs, and omega-3 fatty acids [4, 5], a notable observational study was conducted in Japan in 2001. Hotta et al. reported that tonsillectomy combined with steroid pulse (TSP) therapy was more effective than steroid pulse (SP) monotherapy in achieving clinical remission (disappearance of haematuria and proteinuria) [6]. Subsequently, in Japan, there has been active research on TSP[7–9], and as of 2008, TSP was carried out in approximately 60% of medical facilities nationwide [10]. In the latest web-based survey of members of the Japanese Society of Nephrology, approximately 35% of the respondents reported administering TSP therapy to approximately 70% of patients newly diagnosed with IgAN [11].

Several studies have reported the effectiveness of TSP in improving urinary findings [7, 12–15]. It has also been reported that improvement in urinary findings associated with the effectiveness of TSP therapy is related the duration of the nephropathy which was defined as the period from the first appearance of hematuria to the initiation of TSP therapy [16, 17]. However, in these studies, the treatment regimens for the control groups included various therapies other than SP, including oral steroids and RAS inhibitors. Additionally, the comparison and intervention groups were differentiated based on the presence or absence of tonsillectomies. Differences were also observed in the definitions of clinical remission. Therefore, caution is required when interpreting the research results because of the heterogeneity in comparisons and outcome assessments. However, studies focusing on the suppression of renal function decline as an outcome are limited [12, 18–21], and research results are inconsistent. There are a few reported cases of TSP implementation overseas[22–24], and as a result, there is insufficient evidence supporting the effectiveness of TSP in renal function decline. In the 2021 KDIGO (Kidney Disease: Improving Global Outcomes) guidelines for CGN, glucocorticoids should be individually discussed, considering their risks and benefits. Contrastingly, due to the lack of supporting data worldwide, tonsillectomy is not strongly recommended but would be applied only to Japanese patients [25].

Therefore, the objective of this study was to examine the association between renal prognosis and IgAN when combined with steroid pulse therapy with or without tonsillectomy. While there are few global instances of TSP implementation, and evidence is not firmly established, exploring the correlation between TSP and renal function in Japan, where it is widely adopted, may provide important insights for clinicians worldwide. This exploration may potentially influence the decision-making process of clinicians treating patients with IgAN to determine whether to perform tonsillectomy as part of the treatment strategy.

## Methods

### Study design and setting

For this retrospective cohort study, we used data from the Real World Data (RWD) database, which is managed by the Health, Clinic, and Education Information Evaluation Institute, with support from JMDC Inc. (Tokyo, Japan) [26–29]. It contains records of 24 million patients from 225 medical institutions across Japan as of 2022 and includes the following patient information: demographic data, diagnoses according to the International Classification of Diseases, 10th Revision (ICD-10) codes, procedures, medications, and laboratory test results. This database only contains records from the same facility; thus, records from other facilities during the same period or follow-ups after transfer cannot be accessed. This study was approved by Kyoto University and Graduate School and Faculty of Medicine, Ethics Committee (R3714) and did not require individual consent because the data were anonymized. This study adhered to the principles of the Declaration of Helsinki.

### Patients’ criteria

We extracted data from patients with diagnostic codes for IgAN (ICD-10 code: N028) for whom the Anatomical Therapeutic Chemical (ATC) code for methylprednisolone (H02AB04), 125 mg or more/day intravenously on three consecutive days, had been prescribed within 1 year of the cohort entry date (date when the diagnostic codes for IgAN were first assigned). The cohort entry date was from April 2002 to March 2021. Other inclusion criteria were: age ≥ 18 years at the cohort entry date and an index estimated glomerular filtration rate (eGFR; defined as the eGFR measured close to the cohort entry date [within 90 days]) of over 30 mL/min/1.73 m^2^. We excluded patients without index eGFR data, without available follow-up observation data more than 1 year after the cohort entry date, who had undergone renal replacement therapy before or within 1 year of the cohort entry date, in whom the procedural code for tonsillectomy (K377) was assigned before the cohort entry date, who were prescribed oral corticosteroids or other immune-suppressive agents before the cohort entry date, who had a diagnostic code for lupus nephritis (ICD-10 code: M321) or IgA vasculitis (ICD-10 code: D690) before the cohort entry date, and without proteinuria data for covariates. We used landmark analysis to reduce the immortal time bias[30, 31] because the time between IgAN diagnosis and tonsillectomy differed among patients, and no outcome occurred between diagnosis and treatment in certain patients. We defined the landmark time as one year after IgAN diagnosis, and patients in whom one of the outcomes occurred within the landmark time were excluded from the analysis. Patients were categorized into the TSP and SP groups based on whether the procedural code for tonsillectomy was assigned within 1 year of the IgAN diagnosis. This allocation was performed according to the intention-to-treat analysis regardless of the procedural code for tonsillectomy assigned after the landmark time. The time window for this study is summarized in Supplementary Fig. 1.

**Fig. 1:**
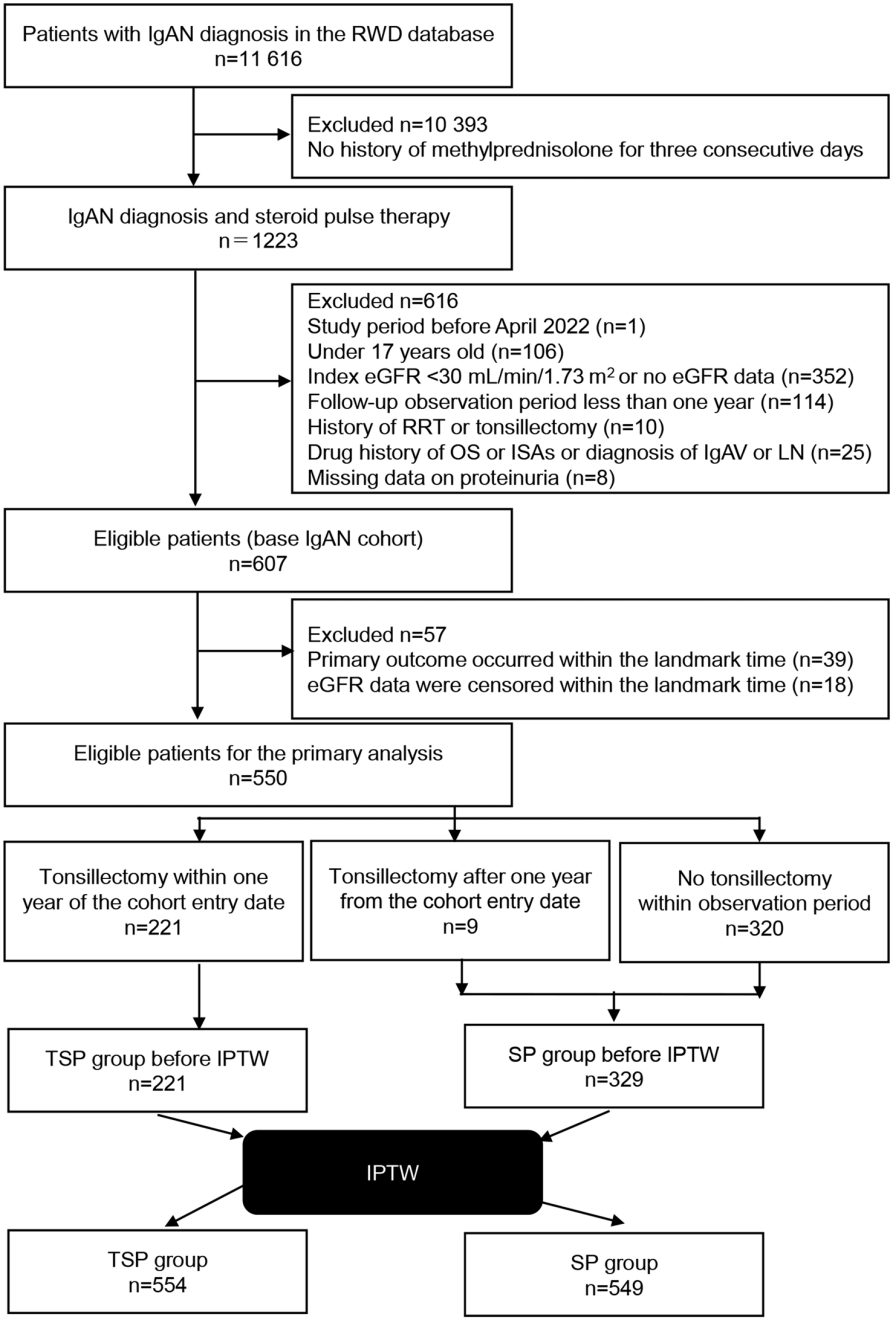
Flow diagram of patients analysed for the primary outcome. IgAN, IgA nephropathy; RWD, the Real World Data; eGFR, estimated glomerular filtration rate; RRT, renal replacement therapy; OS, oral corticosteroids; ISAs, immunosuppressive agents; IgAV, IgA vasculitis; LN, lupus nephritis; TSP, tonsillectomy and steroid pulse; SP, steroid pulse; IPTW, Inverse probability of treatment weighting

### Variables

The primary outcome was the composite outcome of a 30% decline in eGFR from the index eGFR (confirmed twice at least 30 days apart) and the initiation of dialysis. A 30% decline in eGFR is in line with a 30–40% reduction in eGFR over 2–3 years in patients with CKD, serving as a surrogate endpoint for renal failure with replacement therapy [32]. We calculated the eGFR using the following well-validated formula proposed by the Japanese Society of Nephrology [33]:

eGFR (mL/min/1.73 m^2^) = 194 × s-Cre^−1.094^ (mg/dL) × age^−0.287^ (years) × (0.739 for females)

The initiation of dialysis was defined as the addition of the procedure code for permanent dialysis (J038 or J042) in two consecutive months. Secondary outcomes were the composite outcome of end-stage renal failure (eGFR ≤ 15 mL/min/1.73 m^2^, confirmed twice within 30 days) and initiation of dialysis and eGFR slope (mL/min/1.73 m^2^/year) of each patient during the first year after the cohort entry date. To calculate the eGFR slope, we used the index values and the most recent eGFR data measured > 365 days after the cohort entry date. This value indicated annual eGFR loss (mL/min/1.73 m^2^/year) during the first year after diagnosis.

Covariates used included age, sex, laboratory data (eGFR, proteinuria [urine dipstick and random proteinuria measurement], haematuria [urinary occult blood], low-density lipoprotein cholesterol, uric acid, and immunoglobulin A [IgA]), body mass index (BMI), comorbidities (type 2 diabetes), medications (oral hypoglycaemic agents, RAS inhibitors, antihypertensive drugs, lipid-lowering agents, uric acid-lowering agents, and antiplatelet drugs), procedures (tonsillectomy and percutaneous renal biopsy), and the number of hospital beds. Covariates were identified by their diagnostic, procedural, and Anatomical Therapeutic Chemical (for medications) codes (Supplementary Table 1), and the period during which the covariates were extracted is described in Supplementary Fig. 1.

**Table 1:** Baseline characteristics of the study population before and after IPTW for the primary outcome

	Before IPTW			After IPTW		
Variables	SP group	TSP group	SMD	SP group	TSP group	SMD
	n = 329	n = 221		n = 549	n = 554	
**Variables used for PS calculation**						
Age (years), median (IQR)	37 (28–47)	38 (28–47)	0.048	37 (28–47)	38 (28–48)	0.001
Male sex, n (%)	155 (47.1)	101 (45.7)	0.028	255 (46.4)	257 (46.5)	0.001
Index eGFR (mL/min/1.73 m^2^), mean (SD)	73.7 (24.8)	75.4 (23.9)	0.068	74.3 (24.9)	74.5 (23.7)	0.005
Proteinuria^a^, n (%)			0.198			0.017
₋ or ±	44 (13.4)	37 (16.7)		79 (14.4)	77 (13.9)	
1 + or 2 +	210 (63.8)	150 (67.9)		361 (65.8)	363 (65.5)	
3 + or 4 +	75 (22.8)	34 (15.4)		109 (19.9)	114 (20.6)	
Haematuria^b^, n (%)			0.084			0.015
₋ or ±	20 (6.1)	10 (4.5)		31 (5.6)	30 (5.4)	
1 + or 2 +	164 (49.8)	117 (52.9)		280 (51.0)	280 (50.5)	
3 + or 4 +	145 (44.1)	94 (42.5)		238 (43.4)	244 (44.0)	
History of renal biopsy, n (%)			0.145			0.001
-	316 (95.8)	205 (92.3)		514 (93.6)	519 (93.7)	
+	14 (4.3)	17 (7.7)		35 (6.4)	35 (6.3)	
Hospital size by the number of beds, n (%)			0.347			0.016
≥500	181 (55.0)	158 (71.5)		338 (61.6)	337 (60.8)	
<500	148 (45.0)	63 (28.5)		211 (38.4)	217 (39.2)	
Medication use, n (%)						
Antiplatelet agents						
Dipyridamole	5 (1.5)	2 (0.9)	0.056	7 (1.3)	6 (1.1)	0.014
Dilazep hydrochloride hydrate	20 (6.1)	13 (5.9)	0.008	31 (5.6)	29 (5.2)	0.019
Oral hypoglycaemic agents	3 (0.9)	4 (1.8)	0.078	7 (1.3)	7 (1.3)	0.003
RAS-antagonists	92 (28.0)	46 (20.8)	0.167	138 (25.1)	140 (25.3)	0.002
Antihypertensive drugs	46 (13.9)	21 (9.5)	0.14	68 (12.4)	75 (13.5)	0.032
Lipid-lowering agents	38 (11.6)	16 (7.2)	0.148	54 (9.8)	54 (9.7)	0.002
Uric acid-lowering agents	18 (5.5)	17 (7.7)	0.09	33 (6.0)	32 (5.8)	0.013
Comorbidity, n (%)						
Type2 diabetes	52 (15.8)	40 (18.1)	0.061	93 (17.0)	92 (16.6)	0.009
**Variables not used for PS calculation**						
BMI (kg/m^2^), mean (SD)	23.3 (3.6)	23.0 (3.7)	0.1	23.1 (3.6)	23.1 (3.6)	0.007
Missing data, n (%)	251 (76.3)	72 (32.6)		413 (75.2)	170 (30.7)	
Proteinuria^c^ (g/Cre), median (IQR)	0.92 (0.45–1.86)	0.69 (0.32–1.38)	0.306	0.90 (0.43–1.78)	0.68 (0.31–1.42)	0.283
Missing data, n (%)	245 (74.5)	53 (24.0)		409 (74.5)	122(22.0)	
LDL-C (mg/dL), mean (SD)	123 (38)	121 (33)	0.048	123 (38)	122 (33)	0.033
Missing data, n (%)	131 (39.8)	46 (20.8)		219 (39.9)	112(20.2)	
Uric acid (mg/dL), mean (SD)	5.8 (1.6)	5.7 (1.5)	0.041	5.8 (1.6)	5.8 (1.6)	0.019
Missing data, n (%)	2 (0.6)	1 (0.5)		3 (0.5)	3 (0.5)	
IgA (mg/dL), mean (SD)	320 (105)	327 (113)	0.063	320 (106)	326 (114)	0.05
Missing data, n (%)	82 (24.9)	37 (16.7)		127 (23.1)	94 (16.9)	
Observation period^d^ (years), median (IQR)	4.16 (2.65–6.43)	3.16 (1.90–4.60)	0.517	4.11 (2.49–6.43)	3.13 (1.83–4.53)	0.503

^a^ The urine dipstick protein test

^b^ The urinary occult blood test

^c^ The random proteinuria measurement test

^d^ From cohort entry date to the date of the last observation in the database

IPTW, inverse probability of treatment weighting; SP, steroid pulse; TSP, tonsillectomy and steroid pulse; SMD, standardized mean difference; PS, propensity score; IQR, interquartile range; SD, standard deviation; eGFR, estimated glomerular filtration rate; RAS, renin–angiotensin system; BMI, body mass index; LDL-C, low-density lipoprotein cholesterol

### Statistical analysis and ethics approval

Continuous variables are presented as means and standard deviations (SDs) or medians and interquartile ranges (IQR). Categorical variables are presented as frequencies and percentages. Inverse probability of treatment weighting (IPTW) using the propensity score (PS) was used to adjust for patients’ backgrounds between the TSP and SP groups [34]. The PS was estimated using a logistic regression model adjusted for covariates. Covariates used for PS calculation were sex, age, number of hospital beds, index eGFR, proteinuria (urine dipstick), haematuria, comorbidities (type 2 diabetes), medications, and procedures (percutaneous renal biopsy), in reference to previous studies [18–20]. The absolute standardized mean difference (ASMD) was used to compare the two groups’ covariate balance before and after IPTW [35]. Reasonable cutoffs for ASMD ranged from 0.1 to 0.25; hence, we considered ASMD < 0.2 to be well-balanced. To investigate the association between tonsillectomy and composite outcomes, we conducted a Kaplan-Meier survival analysis and weighted Cox regression analysis by applying IPTW to estimate hazard ratios (HRs) and 95% confidence intervals (CIs). Data were censored at death or the date of last observation in the database. The index date was defined as the landmark time, which was 1 year after IgAN diagnosis, and log-rank tests were used for comparison. The incidence rate was estimated using the incidence density method, which involves dividing the overall outcome counts by the overall person-time of all eligible patients during the follow-up period after the landmark time [36]. As for the secondary outcome, we compared the eGFR slope between the two groups using the Student’s t-test. Subgroup analyses were performed for age (18–49 vs. ≥ 50), sex, index eGFR (30–60 vs. > 60), proteinuria ((-), (+-) vs. (+), (2 +) vs. (3 +), (4 +)), and haematuria (grouping similar to proteinuria). In each subgroup, we repeated the IPTW in the same manner as in the main analysis. We conducted two sensitivity analyses to confirm the robustness of our results. First, we changed the landmark time from one year to six months. In this analysis, we selected patients who were prescribed steroid pulse therapy within six months of cohort entry. We excluded patients without available follow-up eGFR data for more than six months after the cohort entry date and those in whom one of the outcomes occurred within six months. Both groups were reclassified in accordance with tonsillectomy within six months of the cohort entry date. Patients who underwent tonsillectomy after the landmark time were assigned to the SP group but were censored at the time of tonsillectomy to avoid considering the effects after tonsillectomy. Second, we conducted propensity score matching instead of IPTW. We used 1:1 matching, a logistic regression model, and the non-replacement nearest-neighbor method with a caliper width of 0.2 of the SD. Subgroup and sensitivity analyses were performed only for the primary outcome. Assuming an event rate of 10%, an expected hazard ratio of 0.33, a significance level of 0.05, and a statistical power of 80%, the required sample size was a total of 213 participants [18, 37].

All P-values were two-sided, and statistical significance was set at P < 0.05. All analyses were performed using R version 4.2.3 (R Foundation for Statistical Computing, Vienna, Austria).

This study was approved by the Ethics Committee of Kyoto University (R3714) and did not require individual consent because the data were anonymized. The need for informed consent was waived by the Ethics Committee of Kyoto University. This study adhered to the principles of the Declaration of Helsinki.

## Results

A total of 11 616 patients were screened for IgAN. The number of eligible patients (base IgAN cohort) was 607, of which 550 were eligible for the primary analysis: 221 and 329 in the TSP and SP groups, respectively (Fig. 1)

In the study population, approximately 40% of patients underwent tonsillectomy within 1 year of IgAN diagnosis (Table 1). There were no differences in sex, age, or index eGFR between the two groups. Urine examination showed no differences in haematuria, but when considering random proteinuria measurements, the SP group exhibited mildly higher proteinuria levels. Both groups had a low rate of renal biopsies, and the TSP group had a higher proportion of hospitals with more than 500 beds. Among the 31 patients who underwent renal biopsy, the biopsy date ranged from − 80 days to + 39 days relative to the diagnosis date of IgA nephropathy, with 5 patients undergoing biopsy after the diagnosis date. The kidney biopsy was likely performed at another facility, and the actual date of the biopsy was unknown. There were no significant differences in medication use between the two groups. Additionally, the median observation period was approximately one year longer in the SP group. (SP group 4.2 vs. TSP group 3.2 (years), P = 0.517) The ASMD of all variables used for PS calculation was < 0.2, and the ASMD of variables not used for PS calculation was < 0.2, after adjusting for other covariates other than random proteinuria measurement. We found that among the study population, one received a daily steroid pulse dose of 125 mg, two received 250 mg, and the remaining received 500 mg or 1000 mg. The number of steroid pulse sessions and the presence or absence of post-maintenance steroid therapy were compared between groups. (Supplementary Table 2) In both groups, more than half of the patients received three courses of steroid pulse therapy, and post-steroid therapy was administered in approximately 80% of the cases.

We excluded patients in whom each outcome occurred within one year of the cohort entry date; therefore, the number of eligible patients differed for secondary outcomes (Supplementary Table 3 and Supplementary Fig. 2).

**Fig. 2:**
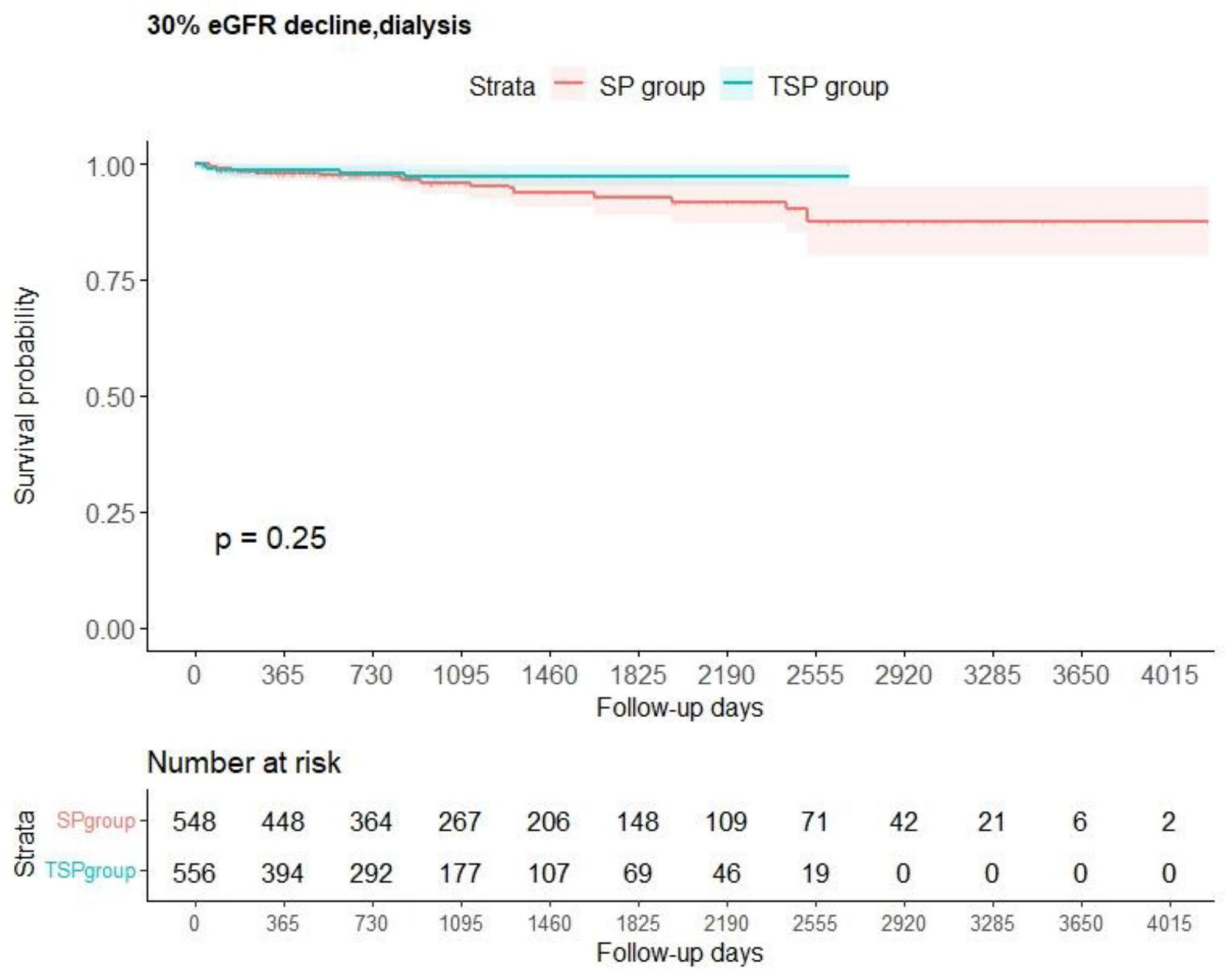
Kaplan–Meier curves for the main analysis of the primary outcome. The survival probability of the primary outcome was calculated using the Kaplan–Meier method. The log–rank test was used to calculate the P value.eGFR, estimated glomerular filtration rate; SP, steroid pulse; TSP, tonsillectomy and steroid pulse

**Fig. 3:**
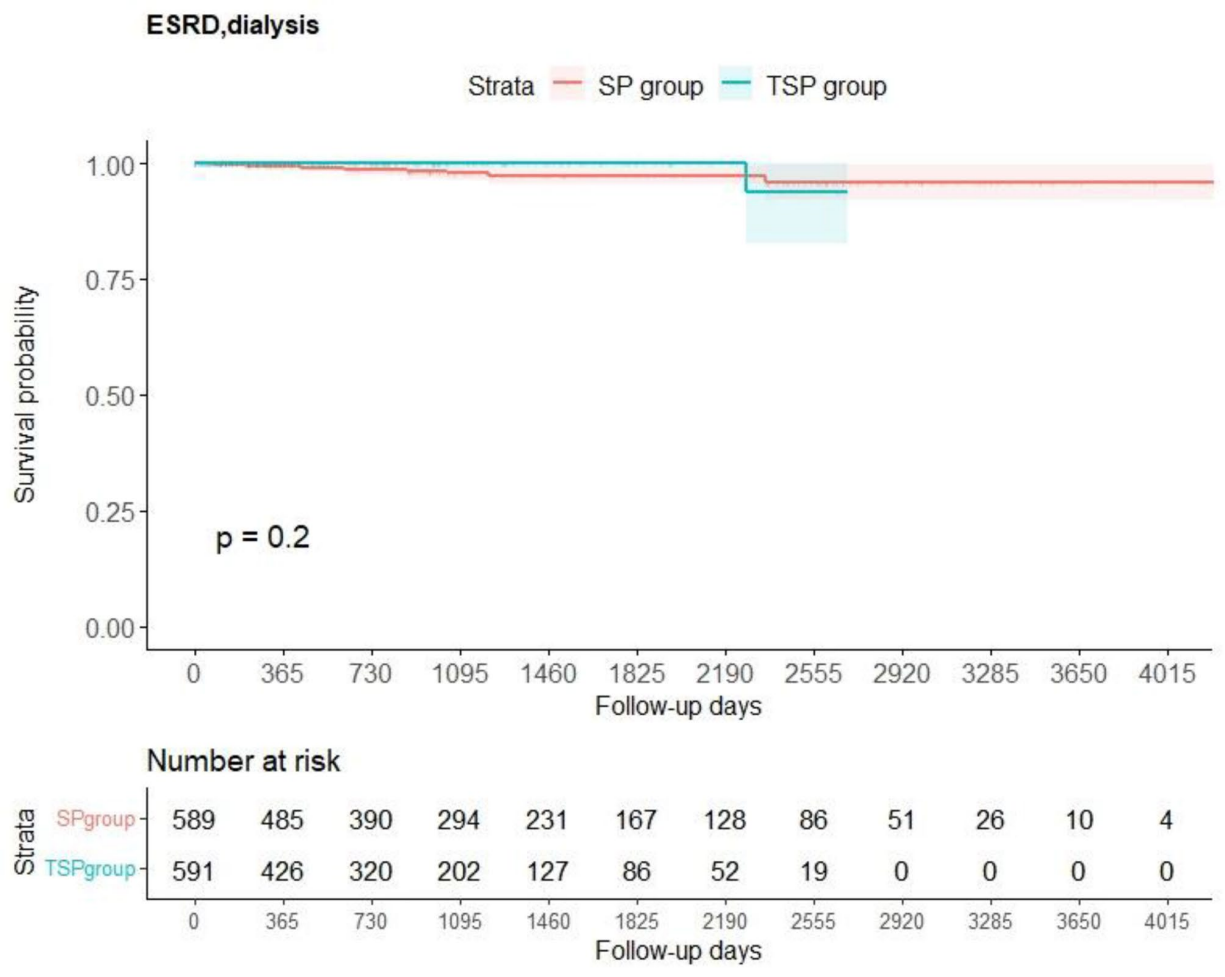
Kaplan–Meier curves for the main analysis of the secondary outcome. The survival probability of the secondary outcome was calculated using the Kaplan–Meier method. The log-rank test was used to calculate the P value. ESRD, end-stage renal failure; SP, steroid pulse; TSP, tonsillectomy and steroid pulse

We plotted the Kaplan–Meier curves of the primary (Fig. 2) and secondary (Fig. 3) outcomes. Furthermore, among the patients in whom the primary outcome occurred, none initiated dialysis before a 30% decrease in eGFR. In the Kaplan–Meier analyses and log–rank tests, the primary outcome did not differ between the groups. (P = 0.25) Incidence ratios of primary outcome per 100 000 person–days were 4.24 (95% CI:2.51-6.70) and 2.50 (95% CI:0.81-5.84) among the SP and TSP groups, respectively (Table 2). The groups did not significantly differ in the primary outcome according to weighted Cox regression analysis by applying IPTW (HR: 0.58 [95% CI: 0.22-1.54], P = 0.28). The secondary outcome also showed no significant difference between the two groups in both log-rank test and weighted Cox proportional hazards regression analysis (HR: 0.35 [95% CI: 0.04-3.43], P = 0.37). There was also no observed difference in the eGFR slope between the two groups after IPTW analysis (eGFR slope after IPTW: SP group -1.30 [mean SD 10.95] vs. TSP group -0.74 [mean SD 10.87] (mL/Fmin/1.73 m2/year), P = 0.565). The subgroup analyses had similar results to the main analyses, but In some subgroups, the number of patients was small, and even after applying IPTW, the ASMD remained 0.2, indicating an imbalance between the two groups in those subgroups (Table 3). The results of the two sensitivity analyses were consistent with those of the main analysis, confirming the robustness of the main analyses.

**Table 2:** Frequencies of events, incidence rate, and hazard ratios (HRs) for primary and secondary outcomes in the main analysis

Outcome	Events	Patients	Person-days	Incidence rate^a^ (95% CI)	Adjusted^b^ HR (95% CI)	P-value
Primary outcome Composite; 30% eGFR decline, dialysis						
SP group	18	329	424581	4.24 (2.51–6.70)	Ref	
TSP group	5	221	199820	2.50 (0.81–5.84)	0.58 (0.22–1.54)	0.28
Secondary outcome Composite; ESRD, dialysis						
SP group	8	359	481272	1.66 (0.72–3.28)	Ref	
TSP group	1	230	216289	0.46 (0.01–2.58)	0.35 (0.04–3.43)	0.37
	Before IPTW			After IPTW		
Secondary outcome	SP group	TSP group	P- value	SP group	TSP group	P- value
	n = 359	n = 230		n = 359	n = 230	
eGFR slope (mL/min/1.73 m^2^/year), mean (SD)	−1.06 (10.96)	−0.74 (10.51)	0.727	−1.30 (10.95)	−0.74 (10.87)	0.565

^a^ Incidence rate per 100 000 person-days

^**b**^ Weighted Cox regression analysis by applying inverse probability of treatment weighting (IPTW) using propensity scores (PS). The covariates used for PS calculation were sex, age, number of hospital beds, index eGFR, proteinuria (urine dipstick), haematuria, type 2 diabetes, medications, and percutaneous renal biopsy.

SP, steroid pulse; TSP, tonsillectomy and steroid pulse; HR, hazard ratio; CI, confidence interval; eGFR, estimated glomerular filtration rate; ESRD, end-stage renal failure

**Table 3:** The number of events and hazard ratios (HRs) for primary outcome in subgroup analysis and sensitivity analysis

		No.events/No.patients	No.events/No.patients		
Subgroup analysis		SP group n = 329	TSP group n = 221	Adjusted^a^ HR (95% CI)	P-value
Age	18–49	14/259	3/180	0.43 (0.13–1.44)	0.17
(years)	≥50	4/70	2/41	0.80 (0.13–4.80)	0.81
Sex	Male	8/155	2/101	0.44 (0.10–2.01)	0.29
	Female	10/174	3/120	0.63 (0.18–2.21)	0.47
Index eGFR	30–60	7/98	3/60	1.08 (0.29–4.03)	0.91
(mL/min/1.73 m^2^)	>60	11/231	2/161	0.34 (0.08–1.48)	0.15
Proteinuria	(-), (+-)	1/44	0/37	-	-
	(+), (2 +)	10/210	3/150	0.53 (0.15–1.86)	0.32
	(3 +), (4 +)	7/75	2/34	0.48 (0.10–2.38)*	0.37
Haematuria	(-), (+-)	1/20	1/10	1.42 (0.11–19.1)*	0.79
	(+), (2 +)	7/164	1/117	0.19 (0.02–1.57)	0.12
	(3 +), (4 +)	10/145	3/94	0.76 (0.22–2.67)	0.67
Sensitivity analysis		No.events/No.patients	No.events/No.patients		
Landmark time change (from one year to six months)		SP group n = 305	TSP group n = 159	Adjusted^a^ HR (95% CI)	P-value
		20/305	5/159	0.51 (0.19–1.38)	0.19
1:1 propensity score matching		SP group n = 203 n = 305	TSP group n = 203 n = 159	Adjusted^a^ HR (95% CI) (95% CI)	P-value
		14/203	5/203	0.47 (0.17–1.33)	0.16

^a^Weighted Cox regression analysis by applying inverse probability of treatment weighting (IPTW) using propensity scores (PS). The covariates used for PS calculation were sex, age, number of hospital beds, index eGFR, proteinuria (urine dipstick), haematuria, type 2 diabetes, medications, and percutaneous renal biopsy

^*^the ASMD of some covariates remained ≥ 0.2 even after applying IPTW, indicating an imbalance between the two groups owing to the number of patients was small SP, steroid pulse; TSP, tonsillectomy and steroid pulse; HR, hazard ratio; CI, confidence interval; eGFR, estimated glomerular filtration rate

## Discussion

In this retrospective cohort study using a nationwide database, we aimed to examine the association between renal prognosis and IgAN when combined with steroid pulse therapy with or without tonsillectomy. Our findings revealed that approximately 40% of patients who met the inclusion criteria underwent tonsillectomy within one year after diagnosis. We also found no significant differences in any of the outcomes between the TSP and SP groups.

Several studies have reported the efficacy of TSP therapy in improving renal function; however, the results have been inconsistent. In Hoshino et al.’s study [19], a multicenter and large-scale cohort with mean follow-up period of 8.3 years was employed to compare outcomes for the initiation of dialysis as an indication of ESRD. The study compared TSP therapy with SP, oral corticosteroid (OS), and renin-angiotensin system inhibition (RAS) alone. The results of the comparison between TSP and SP, both in the primary analysis (adjusted HR: 1.33 [95% CI: 0.44–4.04]) and in the analysis using propensity score matching method (adjusted HR: 1.28 [95% CI: 0.32–5.10]), did not show statistically significant differences. A similarity to this study lies in the straightforward comparison between the TSP and SP groups, where, despite a prolonged follow-up period, no significant differences in outcomes were observed. Although the study population was larger, it included a mixture of other treatment modalities. The actual number of target patients was 209 in the TSP group and 103 in the SP group, which is lower than the number of patients in our study. Additionally, Hirano et al. reported a study using the Japanese Nationwide Retrospective Cohort Study in IgAN (JNR-IgAN cohort), employing a large-scale cohort with mean follow-up period of 5.8 years to set outcomes for the first occurrence of a 1.5-fold increase in serum creatinine level from baseline or dialysis initiation [18]. Here, propensity score matching (PSM) and IPTW methods were employed to balance the background characteristics of the target patients, and both analyses demonstrated the association between tonsillectomy and a lower risk of the primary outcome (adjusted HR using PSM: 0.34 [95% CI: 0.13–0.77], adjusted HR using IPTW: 0.37 [95% CI: 0.17–0.72]). This study, similar to our study, utilized statistical analysis methods such as PMS and IPTW to balance the two groups of patients. Here, the time of renal biopsy was designated as the index date to initiate follow-up of outcomes. However, the exposure status was defined based on the treatment received within one year of renal biopsy. Therefore, the study may not have adequately addressed immortal time bias, and the results may have been overestimated.

Several of our findings are worth discussing. First, we confirmed that approximately 40% of the patients in our study underwent tonsillectomy and that there were no significant differences in patient characteristics between the two groups. In an European multicentre retrospective study [22], the authors noted that the percentage of patients undergoing TSP therapy in the target population was approximately 1.7%, which is quite low. However, similar to previous reports [10, 11, 38], TSP therapy has been confirmed to be prevalent in Japan. In the guidelines for IgA nephropathy in Japan [39], the decision to perform tonsillectomy is left to the discretion of each physician, based on factors such as renal function, proteinuria, haematuria, age, blood pressure, and histological severity. In this study, there were no significant differences between the two groups in terms of renal function, proteinuria, haematuria, age, or use of antihypertensive medication. This suggests that the decision to perform tonsillectomy is likely determined by physicians based on histological severity classification, which served as an unmeasured confounding factor in this study. Second, no significant difference was observed in renal outcomes between the TSP and SP groups. This is primarily attributed to two reasons. The first reason is believed to be an insufficient observation period until events occur. Regarding the annual eGFR decline rate in IgA nephropathy, multiple reports have confirmed that it decreases by − 1 to − 3 mL/min/1.73 m² per year, depending on the initial kidney function and proteinuria level [40, 41]. In other words, since the baseline eGFR of the patients in this study was about 70 mL/min/1.73 m², it would take approximately 6 to 20 years of long-term observation for a 30% eGFR decline event to occur. The study period in the database spanned approximately 20 years, but the median observation period of the target patients was approximately 3–4 years, which was shorter than that in the aforementioned studies. Considering the youthfulness of the patients, low incidence of mortality, and the inherent inability to track post-transfer follow-ups in the database, the most likely cause for this is transfer to another medical facility. Furthermore, since there is no data on urinary findings at the end of the follow-up survey, it is unclear whether the treatment caused remission of the urinary findings and made follow-up unnecessary, or whether it was due to relocation or withdrawal due to the patient’s own self-interruption. Due to the low occurrence of events, this study may have been underpowered to detect the effectiveness of TSP. Assuming an event rate of 5.5%, an expected hazard ratio of 0.58, a significance level of 0.05, and a statistical power of 80%, which was based on the result of our study, the required sample size was a total of 1219 participants. The second reason is the potential for misclassification of the TSP and SP groups. Considering the lower renal biopsy rate and larger bed capacity in the TSP group, it is possible that due to institutional circumstances, tonsillectomy and renal biopsies were performed at other hospitals but not recorded in the database. In reality, these cases might belong to the TSP group but could be misclassified as the SP group, leading to a potential underestimation of the treatment effect. Tonsillectomy is a common surgical technique performed by otolaryngologists. Therefore, if the hospital has an otolaryngologist capable of administering general anaesthesia and performing general surgeries, the likelihood of performing tonsillectomy within the same facility is high, and it is considered unlikely to be performed in other hospitals. There were no previous studies that specifically describe the proportion of IgA nephropathy patients who undergo tonsillectomy and steroid pulse therapy at different facilities, and the extent of patient misclassification remains unclear. Regarding renal biopsy, the maximum interval between the date of diagnosis and the date of renal biopsy among patients who underwent a renal biopsy was within four months, which is a relatively short period. Therefore, we considered the influence of lead-time bias on survival analysis to be minimal. Additionally, we used the presence or absence of renal biopsy as a covariate in the IPTW method, and thus, we also considered the impact of the low biopsy rate on the results to be limited. Considering the above two main reasons, in future research, it is desirable to design studies that allow the tracking of patients even after transfer to ensure long-term observation periods. Third, although the data is limited to one year after treatment, a comparison of the annual eGFR decline slope between the two groups after IPTW showed that the decline was slightly more gradual in the TSP group (−0.74 mL/min/1.73 m²/year) compared to the SP group (−1.30 mL/min/1.73 m²/year). Compared to these previous reports, the SP group showed a decline of − 1.30 mL/min/1.73 m², which is consistent with previous findings, while the TSP group showed a slower decline of − 0.74, mL/min/1.73 m² indicating a slight delay in disease progression. In future studies, it will be necessary to evaluate the continuous eGFR slope, not just the first year after treatment, as a surrogate marker for ESRD in IgA nephropathy.

This study included patients who underwent steroid pulse therapy and tonsillectomy within a certain period after the diagnosis of IgAN. Setting the landmark time at one year after the diagnosis of IgAN and considering it as the starting point for outcome follow-up help address the immortal time bias. In addition, the IPTW method was employed to balance the patient backgrounds, including the use of other treatment drugs. The fact that the missing rate of random proteinuria measurements was similar to that of BMI suggested that BMI was extracted from DPC data, leaving the possibility that random proteinuria measurements were only available for patients with a history of hospitalization. Nevertheless, in any case we considered that random proteinuria measurements had a high rate of missing data and could be supplemented through the qualitative urine protein test, which is used for universal screening in Japan [42]. Hence, the ASMD results indicated that the groups were well balanced. Furthermore, we conducted, to the best of our knowledge, the first study on TSP therapy for IgAN using a nationwide Japanese healthcare record database. Therefore, there is the potential for an evaluation of the effect of tonsillectomy, when combined with steroid pulse therapy, on renal outcomes using real-world data although there are certain limitations due to the information available from the database. In Japan, the combination of tonsillectomy and steroid pulse therapy is common when performing tonsillectomy. Future studies using designs similar to this study, which will evaluate the relationship between tonsillectomy in combination with steroid pulse therapy and renal outcomes, may accumulate evidence. The accumulation of evidence from various studies could assist clinicians in deciding whether to perform tonsillectomy as part of the treatment of IgAN.

Our study has some limitations. First, this was a retrospective cohort study; thus, we are aware of several unmeasured confounding factors such as the previously mentioned renal histological severity classification. Renal biopsy findings are essential to discuss treatment and its effects in the context of IgA nephropathy, but this data could not be extracted from the database. Second, because of the nature of this database, which did not capture medical information from other institutions, there is a possibility of misclassification between the TSP and SP groups if the tonsillectomy was performed at another facility. Similarly, if a renal biopsy is performed at another facility, the exact date of the biopsy may be unknown. Additionally, in the case of patient transfer, follow-up may become impossible. Third, the sample size and observation period in this study may have been insufficient. Fourth, steroid pulse therapy may be used to treat other comorbidities. However, considering that the extraction was limited to cases diagnosed with IgAN within one year and excluded patients with a history of steroids or other immunosuppressive agents in the past, the likelihood of such extraction was considered low. Finally, the accuracy of the ICD-10 code for IgAN has not been validated in Japan.

## Conclusions

We found that approximately 40% of patients underwent tonsillectomy within one year after diagnosis. We also explored the effect of tonsillectomy, when combined with steroid pulse therapy, on renal outcomes using real-world data and found no significant differences in renal outcomes between the TSP and SP groups. This study is likely underpowered due to limitations in both sample size and observation period. It is essential to accumulate research exploring the effectiveness of tonsillectomy in combination with steroid pulse therapy, utilizing databases that can ensure adequate sample sizes and long-term follow-up of over 10 years.

## Electronic supplementary material

Below is the link to the electronic supplementary material.

Supplementary Material 1

## Data Availability

The data that support the findings of this study are available from Real World Data Co., Ltd. (Kyoto, Japan) under license but restrictions apply to the availability of these data, which were used under license for the current study, and so are not publicly available. Data are however available from the authors upon reasonable request and with permission of Real World Data Co., Ltd.
