# Supplementary material for: Association between tonsillectomy plus steroid pulse therapy and renal outcomes in patients with IgA nephropathy: a retrospective cohort study
Source: BMC Nephrol. 2025 Aug 23;26:484. doi: 10.1186/s12882-025-04408-5 (PMC12374458; doi:10.1186/s12882-025-04408-5)
Supplement: Supplementary file 1 — Supplementary Material 1 [file 12882_2025_4408_MOESM1_ESM.docx]

**Supplementary Material**

**Association between tonsillectomy plus steroid pulse therapy and renal outcomes in patients with IgA nephropathy: a retrospective cohort study**

**Supplementary Table 1.** ICD-10, procedural, and ATC medications used to identify covariate codes

**Supplementary Table 2.** Details of steroid pulse regimens

**Supplementary Table 3.** Baseline characteristics of the study population before and after IPTW for secondary outcomes

**Supplementary Figure 1.** Time window of this study

**Supplementary Figure 2.** Study flow of secondary outcomes

**Supplementary Table 1**. ICD-10, procedural, and ATC medications used to identify covariate codes

| **Disease name** | **ICD-10 code** |
| --- | --- |
| IgA nephropathy (IgAN) | N028 |
| Type 2 diabetes mellitus | E11, E12, E13, E14 |
| IgA vasculitis | D690 |
| Lupus nephritis | M321 |
|  |  |
| **Procedure name** | **Procedural code** |
| Tonsillectomy | K377 |
| Percutaneous renal biopsy | D412-02 |
| Permanent dialysis | J038, J042 |
| Kidney transplant | K780-2 |
|  |  |
| **Medication** | **ATC code** |
| methylprednisolone | H02AB04 |
| oral corticosteroid | H02AB01, H02AB02, H02AB04, H02AB06, H02AB09, H02AB10 |
| Immune-suppressive agents |  |
| Cyclophosphamide | L01AA01 |
| Azathioprine | L04AX01 |
| Cyclosporine | L04AD01 |
| Tacrolimus | L04AD02 |
| Mycophenolate mofetil | L04AA06 |
| Mizoribine | L04AX |
| Oral hypoglycaemic agents | A10BA, A10BB, A10BD, A10BF, A10BG, A10BH, A10BJ, A10BK, A10BX |
| RAS antagonists | C09AA, C09CA, C09DA, C09DB, C09DX, C09XA |
| Antihypertensive drugs (Ca blocker and β-blocker) | C08CA, C08DA, C08DB, C08EA, C07AA, C07AB, C07AG |
| Lipid-lowering agents | C10AA, C10AB, C10AC, C10AD, C10AX, C10BA, C10BX |
| Uric acid-lowering agents | M04AA, M04AB, M04AC, M04AX |
| Antiplatelet drugs |  |
| Dipyridamole | B01AC07 |
| Dilazep hydrochloride hydrate | C01DX10 |

ICD-10 code, International Classification of Diseases 10^th^ Revision diagnosis code; ATC code, Anatomical Therapeutic Chemical code; RAS, renin-angiotensin system; Ca, calcium channel

**Supplementary Table 2.** Details of steroid pulse regimens

|  |  | |
| --- | --- | --- |
| Number of steroid pulse sessions, n (%) | SP group  n = 329 | TSP group  n = 221 |
| 1 | 29 (8.8) | 8 (3.6) |
| 2 | 46 (14.0) | 81 (36.7) |
| 3 | 232 (70.5) | 122 (55.2) |
| 4 | 11 (3.3) | 4 (1.8) |
| 5 | 3 (0.9) | 0 (0.0) |
| 6 | 7 (2.1) | 5 (2.3) |
| 7 | 0 (0.0) | 1 (0.5) |
| 8 | 1 (0.3) | 0 (0.0) |
| Maintenance steroid therapy^a^, n (%) | 283 (86.0) | 172 (77.8) |

a. As body weight data could not be extracted from the database, the presence of post-maintenance steroid therapy was defined as the prescription of oral steroids following pulse steroid therapy, regardless of the steroid dosage.

**Supplementary Table 3.** Baseline characteristics of the study population before and after IPTW for secondary outcomes

|  | Before IPTW | | | | After IPTW | | |
| --- | --- | --- | --- | --- | --- | --- | --- |
| Variables | SP group | TSP group | SMD | | SP group | TSP group | SMD |
|  | n = 359 | n = 230 |  | | n = 589 | n = 591 |  |
| **Variables used for PS calculation** |  |  |  | |  |  |  |
| Age (years), median (IQR) | 38 (28–49) | 39 (28–48) | 0.078 | | 38 (28–49) | 39 (29–48) | 0.004 |
| Male sex, n (%) | 167 (46.5) | 105 (45.7) | 0.017 | | 271 (46.0) | 272 (46.0) | 0.001 |
| Index eGFR (mL/min/1.73 m^2^), mean (SD) | 73.1 (25.7) | 75.0 (24.2) | 0.074 | | 73.8 (25.9) | 74.0 (24.0) | 0.008 |
| Proteinuria^a^, n (%) |  |  | 0.204 | |  |  | 0.014 |
| ₋ or ± | 48 (13.4) | 38 (16.5) |  | | 84 (14.3) | 83 (14.0) |  |
| 1+ or 2+ | 225 (62.7) | 155 (67.4) |  | | 381 (64.7) | 381 (64.5) |  |
| 3+ or 4+ | 86 (24.0) | 37 (16.1) |  | | 124 (21.1) | 127 (21.5) |  |
| Haematuria^b^, n (%) |  |  | 0.076 | |  |  | 0.014 |
| ₋ or ± | 23 (6.4) | 11 (4.8) |  | | 35 (5.9) | 33 (5.6) |  |
| 1+ or 2+ | 181 (50.4) | 121 (52.6) |  | | 302 (51.3) | 301 (50.9) |  |
| 3+ or 4+ | 155 (43.2) | 98 (42.6) |  | | 252 (42.8) | 257 (43.5) |  |
| History of renal biopsy, n (%) |  |  | 0.128 | |  |  | 0.007 |
| - | 342 (95.3) | 212 (92.2) |  | | 551 (93.5) | 552 (93.4) |  |
| + | 17 (4.7) | 18 (7.8) |  | | 38 (6.5) | 39 (6.6) |  |
| Hospital size by the number of beds, n (%) |  |  | 0.331 | |  |  | 0.01 |
| ≥500 | 203 (56.5) | 166 (72.2) |  | | 369 (62.7) | 368 (62.3) |  |
| <500 | 156 (43.5) | 64 (27.8) |  | | 220 (37.3) | 223 (37.7) |  |
| Medication use, n (%) |  |  |  | |  |  |  |
| Antiplatelet agents |  |  |  | |  |  |  |
| Dipyridamole | 5 (1.4) | 2 (0.9) | 0.049 | | 7 (1.2) | 6 (1.0) | 0.014 |
| Dilazep hydrochloride hydrate | 22 (6.1) | 13 (5.7) | 0.02 | | 33 (5.6) | 30 (5.1) | 0.023 |
| Oral hypoglycaemic agents | 4 (1.1) | 4 (1.7) | 0.053 | | 8 (1.4) | 8 (1.3) | 0.001 |
| RAS-antagonists | 99 (27.6) | 49 (21.3) | | 0.146 | 148 (25.1) | 148 (25.0) | 0.003 |
| Antihypertensive drugs | 51 (14.2) | 24 (10.4) | | 0.115 | 76 (12.9) | 81 (13.7) | 0.025 |
| Lipid-lowering agents | 40 (11.1) | 17 (7.4) | | 0.13 | 57 (9.7) | 57 (9.6) | 0.004 |
| Uric acid-lowering agents | 21 (5.8) | 18 (7.8) | | 0.078 | 38 (6.5) | 36 (6.2) | 0.013 |
| Comorbidity, n (%) |  |  | |  |  |  |  |
| Type2 diabetes | 59 (16.4) | 45 (19.6) | | 0.082 | 105 (17.8) | 105 (17.8) | 0.005 |
|  |  |  | |  |  |  |  |
| **Variables not used for PS calculation** |  |  | |  |  |  |  |
| BMI (kg/m^2^), mean (SD) | 23.3 (3.6) | 23.0 (3.7) | | 0.083 | 23.0 (3.5) | 23.1 (3.6) | 0.004 |
| Missing data, n (%) | 272 (75.8) | 75 (32.6) | |  | 442 (75.0) | 183 (31.0) |  |
| Proteinuria^c^ (g/Cre), median (IQR) | 1.00  (0.46–2.17) | 0.70  (0.32–1.38) | | 0.328 | 0.94  (0.44–2.13) | 0.69  (0.31–1.44) | 0.304 |
| Missing data, n (%) | 264 (73.5) | 55 (23.9) | |  | 436 (74.0) | 131 (22.1) |  |
| LDL-C (mg/dL), mean (SD) | 125 (46) | 122 (34) | | 0.074 | 125 (45) | 123 (34) | 0.052 |
| Missing data, n (%) | 136 (37.9) | 46 (20.0) | |  | 223 (37.9) | 116 (19.6) |  |
| Uric acid (mg/dL), mean (SD) | 5.8 (1.6) | 5.7 (1.5) | | 0.05 | 5.8 (1.6) | 5.8 (1.5) | <0.001 |
| Missing data, n (%) | 2 (0.56) | 1 (0.43) | |  | 3 (0.5) | 2 (0.3) |  |
| IgA (mg/dL), mean (SD) | 325 (114) | 329 (116) | | 0.03 | 325 (115) | 327 (117) | 0.024 |
| Missing data, n (%) | 84 (23.4) | 38 (16.5) | |  | 127 (21.6) | 98 (16.6) |  |
| Observation period^d^ (years), median (IQR) | 4.11  (2.64–6.42) | 3.17  (1.90–4.67) | | 0.484 | 4.09  (2.50–6.43) | 3.14  (1.84–4.55) | 0.48 |

^a^ Urine dipstick protein test

^b^ The urinary occult blood test

^c^ Random proteinuria measurement test

^d^ From cohort entry date to the date of the last observation in the database

IPTW, inverse probability of treatment weighting; SP, steroid pulse; TSP, tonsillectomy and steroid pulse; SMD, standardized mean difference; PS, propensity score; IQR, interquartile range; SD, standard deviation; eGFR, estimated glomerular filtration rate; RAS, renin-angiotensin system; BMI, body mass index; LDL-C, low-density lipoprotein cholesterol.

**Supplementary Figure 1**. Time window of this study

COV4 (Renal biopsy)

Days [-∞, +90]

COV3 (Comorbidities, medications, BMI)

Days [-180, +0]

COV2 (Baseline laboratory data)

Days [-90, +90] ^a^

COV1 (Age, sex, hospital bed)

Days [-0, +0]

Day0 Cohort entry date

(IgAN diagnosis)

Follow-up window

Days [+366, Censor^d^]

EXPO (Tonsillectomy data)

Days [+0, +365]

EXCL3^c^ (Tonsillectomy data)

Days [-∞, -1]

EXCL2^b^ (RRT data, 30% eGFR decline)

Days [-∞, +365], [+1, +365]

EXCL1 (IgAV or LN, steroid or ISAs)

Days [-∞, -1]

INCL3 (Age ≥18)

Days [-0, +0]

INCL1 (Steroid pulse)

Days [+0, +365]

INCL2 (Index eGFR)

Days [-90, +90] ^a^

Time

^a^ Data closest to the cohort entry date for multiple data points

^b^ Wash-out window for the outcome

^c^ Washout window for exposure

^d^ Death or date of last observation in the database

INCL, inclusion assessment window; EXCL, exclusion assessment window; EXPO, exposure assessment window; COV, covariate assessment window; IgAN, IgA nephropathy; eGFR, estimated glomerular filtration rate; IgAV, IgA vasculitis; LN, lupus nephritis; ISAs, immunosuppressive agents; RRT, renal replacement therapy; BMI, body mass index.

**Supplementary Figure 2**. Study flow of secondary outcomes

TSP group

n=591

SP group

n=589

IPTW

SP group before IPTW

n=359

TSP group before IPTW

n=230

Tonsillectomy within one year of the cohort entry date

n=230

Tonsillectomy after one year from the cohort entry date

n=12

No tonsillectomy

within observation period

n=347

Eligible patients (base IgAN cohort)

n=607

Patients with IgAN diagnosis in the RWD database

n=11 616

IgAN diagnosis and steroid pulse therapy

n=1223

Excluded n=616

Study period before April 2022 (n=1)

Under 17 years old (n=106)

Index eGFR<30 mL/min/1.73 m^2^ or no eGFR data (n=352)

Follow-up observation period less than one year (n=114)

History of RRT or tonsillectomy (n=10)

Drug history of OS or ISAs or diagnosis of IgAV or LN (n=25)

Missing data on proteinuria (n=8)

Excluded n=10 393

No history of methylprednisolone for three consecutive days

No

Excluded n=18

ESRD occurred within the landmark time (n=0)

eGFR data was censored within the landmark time (n=18)

Eligible patients for the secondary analysis

n=589

IgAN, IgA nephropathy; RWD, the Real World Data; eGFR, estimated glomerular filtration rate; RRT, renal replacement therapy; OS, oral corticosteroid; ISAs, immunosuppressive agents; IgAV, IgA vasculitis; LN, lupus nephritis; ESRD, end-stage renal failure; TSP, tonsillectomy and steroid pulse; SP, steroid pulse; IPTW, Inverse probability of treatment weighting.
